# Supplementary material for: Content-rich biological network constructed by mining PubMed abstracts
Source: BMC Bioinformatics. 2004 Oct 8;5:147. doi: 10.1186/1471-2105-5-147 (PMC528731; doi:10.1186/1471-2105-5-147)
Supplement: Additional File 5 — The original Chilibot query results of the term "long-term potentiation (LTP)" and 22 other terms, limiting the latest references analyzed to the years 1990, 1995, 2000, and 2004. [file 1471-2105-5-147-S5.bz2 › chilibotAdditionalFile5/ltp2000/html/PKA_SYNAPTOPHYSIN.html]

 


 **PKA** and **SYNAPTOPHYSIN** 
  
Found 1 abstracts in PubMed,  **1 abstracts were retrieved and analyzed**.  


---

 Search Google  |
 PDF files only 
|  EDU domain only 

---

- Neuron, 2000   **Increasing numbers of synaptic puncta during late phase LTP N cadherin is synthesized, recruited to synaptic sites, and required for potentiation.** .
  It is an open question whether new synapses form during hippocampal LTP.
  Here, we show that late phase LTP L LTP is associated with a significant increase in numbers of synaptic puncta identified by **synaptophysin** and N cadherin, an adhesion protein involved in synapse formation during development.
  During potentiation, protein levels of N cadherin are significantly elevated and N cadherin dimerization is enhanced.
  The increases in synaptic number and N cadherin levels are dependent on cAMP dependent protein kinase **PKA** and protein synthesis, both of which are also required for L LTP.
  Blocking N cadherin adhesion prevents the induction of L LTP, but not the early phase of LTP E LTP .
  Our data suggest that N cadherin is synthesized during the induction of L LTP and recruited to newly forming synapses.
  N cadherin may play a critical role in L LTP by holding nascent pre and postsynaptic membranes in apposition, enabling incipient synapses to acquire function and contribute to potentiation.
